# Emerging Therapeutic Strategies to Overcome Drug Resistance in Cancer Cells

**DOI:** 10.3390/cancers16132478

**Published:** 2024-07-07

**Authors:** Pankaj Garg, Jyoti Malhotra, Prakash Kulkarni, David Horne, Ravi Salgia, Sharad S. Singhal

**Affiliations:** 1 Department of Chemistry, GLA University, Mathura 281406, India; 2 Departments of Medical Oncology & Therapeutics Research, Beckman Research Institute of City of Hope, Comprehensive Cancer Center, National Medical Center, Duarte, CA 91010, USA; 3 Molecular Medicine, Beckman Research Institute of City of Hope, Comprehensive Cancer Center, National Medical Center, Duarte, CA 91010, USA

**Keywords:** drug-resistance mechanisms, targeted therapies, adaptive strategies, biomarker driven approaches, tumor microenvironment, immune checkpoint inhibitors, precision medicine

## Abstract

**Simple Summary:**

This review explores the challenges of drug resistance in cancer treatment and discusses innovative strategies to overcome them. Researchers aim to understand how cancer cells develop resistance to therapies and explore new ways to improve treatment outcomes. They investigate advanced genomic technologies, immunotherapies like CAR-T cells, and targeted therapies that specifically attack cancer cells. By identifying these mechanisms and developing novel approaches, this research aims to enhance the effectiveness of cancer treatments and improve patient survival rates. Insights gained could lead to significant advancements in how we combat drug resistance in cancer, offering hope for better outcomes in the future.

**Abstract:**

The rise of drug resistance in cancer cells presents a formidable challenge in modern oncology, necessitating the exploration of innovative therapeutic strategies. This review investigates the latest advancements in overcoming drug resistance mechanisms employed by cancer cells, focusing on emerging therapeutic modalities. The intricate molecular insights into drug resistance, including genetic mutations, efflux pumps, altered signaling pathways, and microenvironmental influences, are discussed. Furthermore, the promising avenues offered by targeted therapies, combination treatments, immunotherapies, and precision medicine approaches are highlighted. Specifically, the synergistic effects of combining traditional cytotoxic agents with molecularly targeted inhibitors to circumvent resistance pathways are examined. Additionally, the evolving landscape of immunotherapeutic interventions, including immune checkpoint inhibitors and adoptive cell therapies, is explored in terms of bolstering anti-tumor immune responses and overcoming immune evasion mechanisms. Moreover, the significance of biomarker-driven strategies for predicting and monitoring treatment responses is underscored, thereby optimizing therapeutic outcomes. For insights into the future direction of cancer treatment paradigms, the current review focused on prevailing drug resistance challenges and improving patient outcomes, through an integrative analysis of these emerging therapeutic strategies.

## 1. Introduction

Drug resistance in cancer treatment is a complex problem where cancer cells change in ways that help them to avoid being killed by anticancer drugs. This makes it harder to treat or control the growth of tumors [1]. One major cause of drug resistance is when cancer cells develop genetic mutations. These mutations can affect genes that are targeted by drugs, like enzymes or receptors that control cell growth or survival. Also, changes in genes that repair DNA damage can make cancer cells better at fixing drug-induced damage, allowing them to survive and grow even with drug treatment [2]. Additionally, mutations in genes related to cell death pathways can make cancer cells resistant to dying when exposed to drugs, helping them survive against these treatments [3]. Apart from genetic changes, cancer cells can also use sophisticated methods to actively remove drugs from inside themselves. This is carried out through efflux pumps like ATP-binding cassette (ABC) transporters such as P-glycoprotein, which push drugs out of the cell, reducing the amount of drug inside and making them less effective [4]. Moreover, cancer cells can change their characteristics in ways that make them less sensitive to drugs, like becoming more like stem cells or entering a dormant state, which makes them harder to kill with drugs that target fast-growing cells [5].

**Figure 1:**
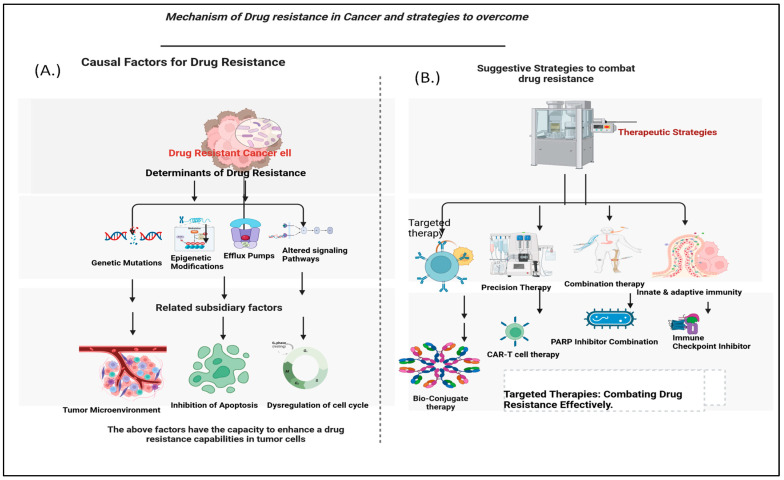
Visual overview: understanding drug resistance mechanisms and innovative strategies for overcoming them. Panel (**A**) details the primary causal determinants contributing to drug resistance, including gene mutations, efflux pumps, alterations in signaling pathways, and tumor heterogeneity. Panel (**B**) showcases a diverse array of advanced therapy modalities aimed at overcoming the detrimental effects of drug resistance in cancer cells, encompassing targeted therapies, immunotherapies, combination treatments, and emerging molecular interventions.

Cancer cells can change their behavior to resist treatments, making it harder to fight the disease. They carry this out by activating survival pathways like PI3K-Akt-mTOR, which helps them survive the stress caused by drugs and grow more. At the same time, they can turn off pathways that would make them expire or stop growing in response to treatment, helping them avoid the harmful effects of the drugs [6]. The environment around the tumor also plays a big role. Areas of low oxygen, called hypoxic areas, can make cancer cells adapt and activate survival pathways, protecting them from dying due to lack of oxygen and making them resistant to certain drugs. The acidic conditions and changes in nutrients in tumors also contribute to drug resistance by favoring the survival of cancer cells that can resist treatment [7]. Changes in how genes are turned on or off, known as epigenetic modifications, also contribute to drug resistance [8]. These changes can silence genes that would normally stop cancer growth or turn on genes that help cancer cells survive treatment. In short, drug resistance in cancer cells is a complex process involving genetic, environmental, and epigenetic factors. Understanding these mechanisms is crucial for developing better treatments that can overcome drug resistance and improve cancer therapy’s effectiveness (Figure 1) [9].

The novelty in overcoming drug resistance in cancer cells lies in the integration of advanced genomic and proteomic profiling, such as Next-Generation Sequencing and Single-Cell Sequencing, to personalize treatments based on individual tumor characteristics. Cutting-edge gene editing technologies like CRISPR-Cas9, RNA interference for targeted gene silencing, and proteomic analysis. offer precise control and insights into resistance mechanisms. High-Throughput Screening accelerates the discovery of effective drug combinations, while 3D culture models like organoids provide realistic tumor environments for better preclinical testing. Nanotechnology enhances drug delivery, overcoming efflux pump issues, and epigenetic therapies reverse resistance-associated changes. Innovations in immunotherapy, including checkpoint inhibitors and CAR-T cell therapy, harness the immune system to target resistant cells. Adaptive and combination therapies strategically prevent and combat resistance, supported by real-time monitoring via liquid biopsies. Artificial Intelligence and Machine Learning analyze complex data for optimized treatment strategies, and biomarker development aids in predicting responses and personalizing treatments [5,6,7,8]. Together, these approaches represent a multifaceted, cutting-edge strategy to improve cancer treatment outcomes and manage drug resistance effectively.

## 2. Drug Resistance’s Impact on Current Cancer Therapies and Patient’s Outcome

Drug resistance is a big problem in cancer treatment, making it hard for treatments to work well. One major issue is multidrug resistance, where cancer cells become insensitive to many different types of anticancer drugs at once. This often happens because cells increase the activity of drug transporters, like P-glycoprotein, which actively push drugs out of the cells [10]. This lowers the amount of drug inside the cells, making them less effective. Also, cancer cells can become resistant through genetic changes that affect drug targets or important pathways for drug action. P-glycoprotein (P-gp), multidrug resistance proteins (MRPs), and breast cancer resistance protein (BCRP) are key players in the development of multidrug resistance (MDR) in cancer cells. These proteins belong to the ATP-binding cassette (ABC) transporter family and actively transport a wide range of anticancer drugs out of cells, reducing their intracellular concentrations and effectiveness. P-gp is one of the most well-studied MDR transporters. It pumps various chemotherapeutic agents out of cancer cells, including doxorubicin, vinblastine, and paclitaxel. P-gp is highly expressed in many types of cancer, contributing significantly to the failure of chemotherapy [2,10]. Multidrug resistance proteins (MRPs) are another important group of efflux transporters. MRPs, such as MRP1 and MRP2, transport a variety of substrates, including organic anions, glutathione conjugates (GS-Es), and drugs like methotrexate and vincristine. MRPs utilize ATP hydrolysis to expel these compounds from the cells, thus lowering their cytotoxic effects on cancer cells [1,4]. Breast cancer resistance protein (BCRP), also known as ABCG2, is another critical transporter implicated in MDR. BCRP is highly expressed in breast cancer and other malignancies. It transports drugs such as mitoxantrone, topotecan, and flavopiridol out of cancer cells, contributing to their resistance to these treatments. Understanding the roles of P-gp, MRPs, and BCRP in drug resistance is essential for developing strategies to overcome MDR in cancer [6,10]. Research is ongoing to identify inhibitors of these transporters, develop drugs that are not substrates for these proteins, and use nanotechnology to bypass these efflux mechanisms, thereby enhancing the efficacy of chemotherapy and improving patient outcomes.

Genetic changes in genes that drugs target, such as tyrosine kinases in targeted therapies, can make these treatments ineffective. Changes in how DNA is repaired can also reduce the impact of chemotherapy drugs by helping cancer cells repair damage more easily [11]. Another challenge is the diversity of cells within tumors. Different areas of a tumor can have different genetic and physical characteristics. This diversity can lead to the emergence of cell populations that are resistant to drugs, making it harder to treat the entire tumor. The environment around tumors also plays a role. Factors like low oxygen, acidity, and changes in nutrients can create conditions that support the survival of drug-resistant cancer cells while making it harder for treatments to work effectively [12].

A crucial factor contributing to the drug resistance and aggressive behavior of cancer cells is the hypoxic environment within the tumor niche. Hypoxia-inducible factor-alpha (HIF-α) plays a pivotal role in this process. Under low-oxygen conditions, HIF-α is stabilized and activates the transcription of various genes that promote angiogenesis, metabolic reprogramming, and survival pathways. This adaptation to hypoxia not only supports tumor growth but also leads to the acidification of the tumor microenvironment, creating a hostile environment for normal cells and enhancing the resistance of cancer cells to therapy [7,8,9]. The acidified environment can inactivate certain drugs and promote the selection of more aggressive cancer cell phenotypes. Additionally, hypoxia drives a metabolic switch from oxidative phosphorylation to glycolysis, even in the presence of oxygen, a phenomenon known as the Warburg effect. This switch to anaerobic respiration under hypoxic conditions is associated with the manifestation of the mutator phenotype, which increases genetic instability and drives cancer progression. It also induces cancer cell mobility, contributing to metastasis. In this context, hypoxia-activated prodrugs (HAPs), also known as bioreductive drugs, have been developed to exploit the hypoxic conditions within tumors. These prodrugs are designed to be activated specifically in low-oxygen environments, thereby selectively targeting hypoxic cancer cells, and minimizing damage to normal tissues [7]. By focusing on the unique metabolic and microenvironmental characteristics of hypoxic tumors, HAPs offer a promising strategy to enhance the efficacy of cancer treatments and overcome resistance.

Drug resistance greatly impacts patient outcomes and survival rates in various ways. Initially, it leads to treatment failure, where tumors that previously responded to therapy become resistant, causing the disease to progress and spread. This not only reduces the patients’ quality of life but also makes it harder to find effective follow-up treatments due to cross-resistance. Drug resistance is also involved in therapeutic relapse, where tumors return after a period of improvement. This recurrence often involves more aggressive cancer cells that resist treatment, making future treatment plans more complex and lowering overall survival rates [13]. Additionally, drug resistance shortens the duration of treatment responses, leading to shorter periods of progression-free survival and overall survival rates. Patients might respond well to treatment at first but then see their disease worsen quickly as drug-resistant cells take over. Moreover, drug resistance brings economic challenges, as developing and using new drugs to fight resistance can be costly. This financial strain affects healthcare systems and puts pressure on patients and their families, especially in areas where advanced cancer treatments are limited [14]. In conclusion, drug resistance is a significant barrier in cancer therapy, affecting patient outcomes, survival rates, treatment effectiveness, and healthcare costs. Overcoming this challenge requires a comprehensive approach involving innovative treatment strategies, personalized medicine, and a deeper understanding of the molecular processes driving drug resistance in cancer cells.

## 3. Progress in Molecular Biology for Unraveling Drug Resistance Mechanisms

Recent advancements in molecular biology have revolutionized our understanding of drug resistance mechanism in cancer, giving us profound insights into the complex interactions among cellular pathways and genetic changes driving this issue. One major area of progress involves uncovering the molecular pathways linked to drug removal from cells [15]. Several studies have identified specific ATP-binding cassette (ABC) transporters like P-glycoprotein (P-gp), multidrug resistance-associated protein (MRP), and breast cancer resistance protein (BCRP), that actively pump drugs out of cancer cells, reducing drug levels inside the cells and leading to multidrug resistance (MDR) [16,17]. Furthermore, improvements in genomics and proteomics have made it easier to spot genetic mutations and changes associated with drug resistance. For instance, mutations in genes that drugs target, such as tyrosine kinases in targeted therapies or topoisomerases in chemotherapy, can cause resistance by altering how drugs bind or affect signaling pathways [18].

The role of epigenetic changes like DNA methylation and histone acetylation in controlling gene expression linked to drug resistance has gained significant attention. These modifications can change how genes involved in drug metabolism, DNA repair, and cell death respond to treatments [19]. Moreover, advancements in single cell sequencing technologies have unveiled the diversity within tumors, identifying cell subsets that are naturally resistant or have developed resistance to specific drugs. This improved understanding of tumor diversity has led to personalized medicine approaches, tailoring treatments based on individual patient traits and molecular markers of drug resistance. Similarly, advanced imaging techniques like positron emission tomography (PET) and magnetic resonance imaging (MRI), along with molecular probes targeting drug resistance markers, allow for the non-invasive monitoring of treatment responses and the emergence of resistant cell populations in real-time [20]. These recent breakthroughs in molecular biology have not only deepened our understanding of drug resistance mechanisms but also paved the way for innovative treatment strategies aimed at overcoming resistance, improving patient outcomes, and advancing cancer therapy.

## 4. Molecular Pathways Involved in Drug Resistance in Cancer

### 4.1. Inhibition of Apoptosis

Apoptosis, known as programmed cell death, plays a critical role in cellular self-destruction triggered by various stimuli, including those provoked by anticancer medications. The Bcl-2 family of proteins stands out as a significant player in the pathway responsible for inhibiting apoptosis [21]. These proteins govern the intrinsic apoptotic process by regulating the permeability of the mitochondrial membrane and the discharge of pro-apoptotic elements like cytochrome C. Cancer cells frequently boost the expression of anti-apoptotic Bcl-2 family proteins (e.g., Bcl-2, Bcl-xL) or reduce the levels of pro-apoptotic counterparts (e.g., Bax, Bak), thereby developing resistance to drugs that induce apoptosis [22]. Additionally, cancer cells may activate survival signaling routes, such as the PI3K-Akt pathway, which phosphorylates and deactivates pro-apoptotic proteins like Bad and caspase-9, ultimately fostering cell survival and resistance to apoptosis [23].

### 4.2. Mechanisms of DNA Repair

DNA damage commonly arises as a consequence of anticancer treatments, including chemotherapy and radiation. Cancer cells employ diverse mechanisms for DNA repair to combat drug-induced DNA damage, thereby enhancing their survival and resistance to therapy. A pivotal pathway involved in DNA repair is the homologous recombination (HR) process, responsible for mending double-strand breaks (DSBs) in DNA [24]. Cancer cells bearing deficiencies in HR, such as those with mutations in BRCA1 or BRCA2 genes, tend to be vulnerable to DNA-damaging agents like platinum-based drugs. Conversely, cancer cells might escalate the levels of DNA repair enzymes, such as poly (ADP-ribose) polymerase (PARP), to boost DNA repair capabilities and confer resistance to drugs targeting DNA [25]. Furthermore, the nucleotide excision repair (NER) pathway and the base excision repair (BER) pathway contribute to rectifying diverse forms of DNA damage induced by chemotherapeutic agents [26].

### 4.3. Dysregulation of the Cell Cycle

The cell cycle undergoes precise regulation via a sequence of checkpoints ensuring accurate cell division and proliferation. Aberrations in cell cycle checkpoints can result in uncontrolled cell proliferation and resistance to drugs targeting the cell cycle. The G1/S checkpoint stands as a critical checkpoint managed by cyclin-dependent kinases (CDKs) and cyclins [27]. Cancer cells might exhibit heightened expression of cyclin D1 or CDK4/6, causing overstimulation of the G1/S checkpoint and resistance to CDK4/6 inhibitors [28]. Similarly, modifications in the p53 pathway, a crucial regulator of cell cycle arrest and apoptosis, can confer resistance to DNA-damaging agents by disrupting cell cycle checkpoints and apoptotic processes [29]. Moreover, cancer cells could bypass the mitotic spindle checkpoint, provoking chromosomal instability and resistance to drugs targeting microtubules like taxanes and vinca alkaloids [30]. In essence, the development of drug resistance in cancer entails intricate molecular pathways that support cell survival, impede apoptosis, mend DNA damage, and disturb the cell cycle. Targeting these pathways and comprehending the mechanisms underpinning drug resistance are imperative for formulating efficacious therapeutic approaches to surmount resistance and enhance treatment outcomes for cancer patients [31].

### 4.4. Efflux Mechanisms and Alternative Pathways

Efflux mechanisms are a significant factor in the development of drug resistance in cancer cells. These mechanisms involve the active transport of anticancer drugs out of the cells, reducing their intracellular concentration and thereby diminishing their effectiveness [31]. The primary proteins involved in drug efflux are the ATP-binding cassette (ABC) transporters, which include P-glycoprotein (P-gp), multidrug resistance-associated proteins (MRPs), and breast cancer resistance protein (BCRP). These transporters use energy from ATP hydrolysis to pump drugs out of the cells, effectively decreasing drug accumulation and facilitating resistance. To combat these efflux mechanisms, researchers are exploring alternative pathways and strategies. One such approach is the development of inhibitors that can block the function of efflux pumps, thereby restoring the intracellular concentration of anticancer drugs. For example, inhibitors targeting P-gp, such as tariquidar and elacridar, have been studied to enhance the efficacy of chemotherapy [16,31]. Another strategy involves using nanoparticle-based drug delivery systems that can bypass the efflux pumps and directly release the drug inside the cancer cells. These nanoparticles can be engineered to evade recognition by efflux transporters, ensuring higher drug retention within the cells. Additionally, combination therapies that include drugs targeting multiple pathways can be employed to reduce the likelihood of resistance. For instance, combining efflux pump inhibitors with standard chemotherapy drugs can enhance overall treatment efficacy and overcome resistance mechanisms [16,17,23,31].

## 5. Targeted Therapies: Combating Drug Resistance Effectively

**Figure 2:**
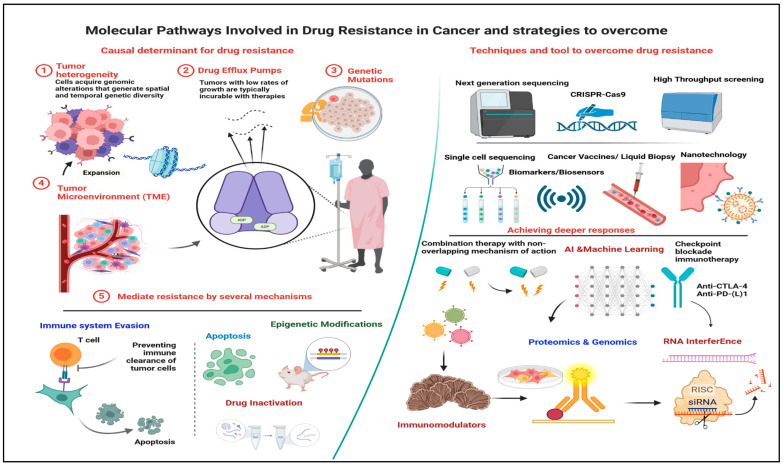
Deciphering molecular pathways of drug resistance in cancer cells: pioneering strategies for conquering resistance challenges, unraveling causal determinants versus harnessing advanced tools and techniques for resisting drug resistance.

Targeted therapies represent a major breakthrough in cancer treatment. They offer a personalized approach by focusing on the specific molecular abnormalities and pathways that drive cancer growth. Unlike traditional chemotherapy, which affects both cancerous and healthy cells, targeted therapies are designed to specifically block or interfere with the molecules or pathways that are unusually active in cancer cells [32]. This idea is based on the understanding that cancer is not a single disease but a collection of different types, each with unique genetic changes and signaling pathways. One of the main advantages of targeted therapies is their ability to overcome drug resistance, a common problem that reduces the effectiveness of traditional treatments [33]. By directly targeting the molecules responsible for drug resistance, these therapies can bypass or counteract the resistance mechanisms used by cancer cells, making treatments more effective again. Numerous strategies help targeted therapies overcome drug resistance (Figure 2).

The commonly used methods of targeted therapy include the following:

1. **Tyrosine Kinase Inhibitors (TKIs):** These drugs inhibit the activity of specific tyrosine kinases, which are enzymes involved in the signaling pathways that regulate cell division and survival. Examples include imatinib (Gleevec) for chronic myeloid leukemia and erlotinib (Tarceva) for non-small cell lung cancer NSCLC).

2. **Monoclonal antibodies**: Monoclonal antibodies are designed to bind to specific antigens on the surface of cancer cells, marking them for destruction by the immune system or blocking growth signals. Examples include trastuzumab (Herceptin) for HER2-positive breast cancer and rituximab (Rituxan) for certain types of lymphoma.

3. **PARP inhibitors**: These inhibitors target the poly (ADP-ribose) polymerase (PARP) enzyme, which is involved in DNA repair. By inhibiting PARP, these drugs induce cancer cell death, particularly in cells with defective DNA repair mechanisms such as BRCA-mutated cancers. Examples include olaparib (Lynparza) and niraparib (Zejula).

4. **mTOR inhibitors**: These drugs inhibit the mammalian target of rapamycin (mTOR), a key protein involved in cell growth, proliferation, and survival. Examples include everolimus (Afinitor) and temsirolimus (Torisel).

5. **Proteasome inhibitors**: Proteasome inhibitors block the proteasome’s function, leading to the accumulation of damaged proteins and inducing cell death, particularly in cancer cells. Examples include bortezomib (Velcade) and carfilzomib (Kyprolis).

6. **BRAF and MEK inhibitors**: These inhibitors target the BRAF and MEK proteins in the MAPK/ERK signaling pathway, which is often mutated in cancers such as melanoma. Examples include vemurafenib (Zelboraf) and dabrafenib (Tafinlar) for BRAF, and trametinib (Mekinist) for MEK.

7. **Angiogenesis inhibitors**: These drugs inhibit the formation of new blood vessels (angiogenesis) that tumors need to grow. Examples include bevacizumab (Avastin) and thalidomide.

8. **Immunotherapies**: Immunotherapies, such as checkpoint inhibitors and CAR-T cell therapy, enhance the body’s immune response against cancer cells. Examples include pembrolizumab (Keytruda) and nivolumab (Opdivo) for checkpoint inhibitors, and CAR-T cell therapies like tisagenlecleucel (Kymriah) and axicabtagene ciloleucel (Yescarta).

By precisely targeting and blocking specific proteins or pathways that are disrupted in cancer cells, these therapies achieve strong anti-tumor effects while sparing normal cells [34]. Overcoming resistance involves blocking alternative or compensatory pathways that cancer cells use to escape conventional treatments, thereby restoring drug sensitivity. Combination therapies enhance anticancer effects and overcome resistance by simultaneously targeting multiple signaling pathways [35]. Personalized medicine tailors’ treatments are based on the unique molecular profile of each patient’s tumor, maximizing effectiveness, and minimizing exposure to ineffective treatments [36]. Adaptive strategies involve continuously monitoring treatment response and molecular changes, allowing for quick identification and adjustment to emerging resistance mechanisms. Despite their benefits, targeted therapies face challenges like acquired resistance and tumor heterogeneity, requiring ongoing research and innovation to improve their effectiveness. In summary, targeted therapies offer a powerful weapon against cancer by focusing on molecular vulnerabilities [37]. Their potential to overcome drug resistance lies in their precision, the synergistic effects of combination therapies, personalized treatment strategies, and adaptive methods. Continued advancements in understanding cancer biology and drug resistance will enhance the role of targeted therapies in improving patient outcomes and advancing cancer treatment [38].

## 6. Precision in Targeted Therapies: Overcoming Cancer Drug Resistance

Targeted therapies are recognized as a powerful tool in the fight against cancer by precisely targeting molecular vulnerabilities within cancer cells. Their effectiveness in overcoming drug resistance is attributed to their precision, ability to counter resistance mechanisms, synergistic effects in combination treatments, personalized therapeutic strategies, and adaptive approaches [39]. As our understanding of cancer biology and drug resistance deepens, targeted therapies are expected to play a crucial role in advancing cancer care and improving patient outcomes. A comprehensive exploration of the varied roles of targeted therapies in combating drug resistance is given below [40].

### 6.1. Tyrosine Kinase Inhibitors (TKIs)

Tyrosine kinases, critical enzymes governing cell signaling pathways that regulate cell growth, division, and survival, are often exceptionally active in various cancers, leading to treatment resistance. Targeted kinase inhibitors (TKIs) are specialized therapies designed to block specific tyrosine kinases, disrupting downstream signaling that fuels cancer advancement [41]. Notable examples include drugs like imatinib (Gleevec), which targets the BCR-ABL fusion protein in chronic myeloid leukemia (CML), and erlotinib (Tarceva), acts on the epidermal growth factor receptor (EGFR) in non-small cell lung cancer (NSCLC). By directly addressing mutated or overactive tyrosine kinases that drive resistance, TKIs can overcome drug resistance, such as those arising from activating mutations within EGFR or BCR-ABL [42].

### 6.2. PARP Inhibitors

PARP inhibitors, a type of targeted therapy, block PARP enzymes that are essential for DNA repair processes, particularly in the base excision repair (BER) pathway. These inhibitors work well in cancers that have flaws in homologous recombination (HR) DNA repair, especially those caused by BRCA1 or BRCA2 mutations [43]. By using the idea of synthetic lethality, PARP inhibitors specifically target cancer cells with HR deficiencies, causing a buildup of DNA damage and cell death. Drugs like olaparib (Lynparza) and rucaparib (Rubraca) combat drug resistance by exploiting the inherent DNA repair faults in cancer cells, making them highly effective in BRCA-mutated breast and ovarian cancers [44].

### 6.3. Immunotherapies (e.g., CAR-T Cell Therapy)

Immunotherapies are revolutionizing cancer treatment by harnessing the immune system to fight and eliminate cancer cells. Chimeric Antigen Receptor (CAR) T cell therapy, a type of immunotherapy, involves modifying a patient’s T-cells genetically to express chimeric antigen receptors [45]. This modification empowers the T-cells to recognize and attack specific proteins on cancer cells. CAR-T cell therapy has shown impressive success in treating blood cancers like certain leukemias and lymphomas. One tactic used by CAR-T cell therapy to overcome drug resistance is through targeting antigens that are not affected by standard treatments or by evading immune evasion mechanisms used by cancer cells [46]. Notably, CAR-T cells targeting CD19 have proven effective in defeating drug resistance in relapsed or refractory B-cell malignancies. 

## 7. Strategic Solutions: Combating Drug Resistance with Combined Treatments

The idea of blending different treatments to tackle drug resistance in cancer is based on the concept that various therapies can be combined to fight resistance, boost treatment effectiveness, and improve patient responses. This strategy recognizes that cancer cells can become resistant to a single treatment method due to various molecular processes [47]. Therefore, using a combination of treatments can target multiple weaknesses simultaneously. The rationale for combining treatments encompasses several compelling reasons (Table 1).

### 7.1. Targeting Diverse Pathways

Cancer cells often employ multiple mechanisms to evade the effects of therapeutic agents. Combining treatments that target distinct molecular pathways or cellular processes increases the likelihood of overcoming resistance [48]. For example, a combination of a TKI targeting a specific signaling pathway and an immunotherapy agent activating the immune system can synergistically target both cancer cell proliferation and immune evasion mechanisms [49].

### 7.2. Synergistic Effects

Some drug combinations exhibit synergistic effects, where the combined action of two or more drugs is greater than the sum of their individual effects. This synergy can lead to enhanced anti-tumor activity, increased apoptosis, and decreased the likelihood of developing drug resistance. For instance, combining a DNA-damaging agent with a PARP inhibitor can induce synthetic lethality in cancer cells with DNA repair deficiencies, such as those harboring BRCA mutations [50].

### 7.3. Overcoming Adaptive Resistance

Cancer cells can adapt and develop resistance to single-agent therapies over time. Combining treatments with different mechanisms of action can prevent or delay the emergence of resistance by targeting multiple vulnerabilities within the tumor cell population. This approach hinders cancer cells’ ability to evolve adaptive resistance mechanisms, leading to more durable treatment responses [51].

### 7.4. Reducing Side Effects:

Combining lower doses of multiple drugs with different toxicity profiles can minimize side effects and toxicity compared to high doses of single agents. This therapeutic strategy, known as dose reduction or dose optimization, aims to achieve maximum efficacy while maintaining manageable side effects, improving patient tolerance and quality of life during treatment [52].

## 8. Efficacious Therapeutic Combinations: From Clinical Trials to Preclinical Studies

The realm of successful treatment combinations that exhibit promise in clinical trials or preclinical studies extends a to broad spectrum of therapeutic approaches. Exploring some notable examples sheds light on the potential of these combinations to revolutionize cancer treatment.

### 8.1. Chemo-Immunotherapy Combinations

Combining chemotherapy drugs with immunotherapy, specifically immune checkpoint inhibitors like pembrolizumab and nivolumab, has proven highly effective in treating a range of cancers such as melanoma, lung cancer, and bladder cancer. These combinations use chemotherapy to trigger a type of cell death that boosts the immune system’s response against cancer cells [53]. Clinical trials have shown significant improvements in survival rates and treatment response with this approach across different cancers. For instance, in melanoma, using dacarbazine alongside ipilimumab (an anti-CTLA-4 antibody) led to better outcomes than using dacarbazine alone [54]. Currently the research is focused on refining treatment schedules, finding biomarkers to select the right patients, and testing new combinations of chemotherapy and immunotherapy to broaden treatment choices.

### 8.2. Targeted Therapy Combinations

The combined strength of targeted therapies with different action mechanisms shows potential in overcoming resistance found in cancers with specific genetic changes. For example, when BRAF and MEK inhibitors are used together in BRAF-mutant melanoma or when EGFR and HER2 inhibitors are combined in HER2-positive breast cancer, there are better treatment responses and a slower development of resistance [55,56]. Clinical trials have revealed encouraging results in patients with these genetic changes. In HER2-positive breast cancer, adding pertuzumab (a HER2-targeted antibody) and chemotherapy to trastuzumab significantly extended progression-free survival compared to using trastuzumab alone [57]. Progress in therapies guided by biomarkers and precision medicine is leading to the investigation of new targeted therapy combinations customized to each patient’s genetic make-up.

### 8.3. PARP Inhibitor Combinations

PARP inhibitors have smoothly integrated into treatment plans alongside chemotherapy, targeted therapies, and immunotherapies for cancers with DNA repair deficiencies, such as ovarian and breast cancers with BRCA mutations. These combinations take advantage of synthetic lethality, causing more DNA damage in cancer cells and leading to better outcomes [58]. The use of PARP inhibitors with other treatments has been highly effective in ovarian and breast cancers. For instance, combining olaparib (a PARP inhibitor) with bevacizumab (an anti-angiogenic agent) improved progression-free survival for patients with BRCA-mutated ovarian cancer [59]. Current research is expanding the use of PARP inhibitors to other DNA repair-deficient cancers and investigating new combinations with targeted therapies and immune-therapies [60].

### 8.4. Dual Immunotherapy Combinations

Combining different immune checkpoint inhibitors or pairing an immune checkpoint inhibitor with other immune-modulating agents, like cytokines or targeted therapies, shows promise in enhancing anti-tumor immune responses. This strategy has proven effective in improving long-term survival rates in cases of melanoma and renal cell carcinoma [61]. Clinical trials have demonstrated durable responses and improved survival rates in melanoma, lung cancer, and renal cell carcinoma with dual immunotherapy combinations [62]. For example, combining nivolumab (anti-PD1) with ipilimumab (anti-CTLA4) in melanoma has shown better outcomes than using either drug alone [63]. Current research aims to optimize dosing regimens, identify biomarkers to predict response, and explore novel immunotherapy combinations, including those with targeted therapies and adoptive cell therapies. The rationale behind combining different treatment modalities to combat drug resistance in cancer is to target multiple pathways, achieve synergistic effects, counteract adaptive resistance, and reduce side effects [64,65]. Success stories of chemo-immunotherapy, targeted therapy combinations, PARP inhibitor synergies, and dual immunotherapy highlight the potential for innovative and impactful treatment strategies in cancer management [66]. 

## 9. Role of the Immune System in Combating Drug-Resistant Cancer Cells

The immune system plays a crucial role in combating drug-resistant cancer cells through a complex interplay of innate and adaptive immune responses that recognize and eliminate cancerous cells. The immune system’s ability to recognize and target cancer cells is based on the recognition of specific antigens or markers expressed by tumor cells, which distinguishes them from normal cells [67]. Several key components of the immune system contribute to combating drug-resistant cancer cells.

### 9.1. Immune Surveillance

The immune system constantly surveys the body for abnormal or mutated cells, including drug-resistant cancer cells. Immune cells such as natural killer (NK) cells, dendritic cells, and macrophages play a vital role in recognizing and eliminating cancer cells that display altered or abnormal antigens [68]. This process of immune surveillance helps detect and eliminate drug-resistant cancer cell populations before they can proliferate and establish resistance mechanisms.

### 9.2. Cytotoxic T lymphocytes (CTLs)

CTLs are a type of immune cell that directly recognizes and kills cancer cells by recognizing specific antigens present on the surface of tumor cells. CTLs play a crucial role in combating drug-resistant cancer cells by targeting and destroying cells that have developed resistance to conventional therapies [69]. Additionally, CTLs can target neoantigens, which are unique antigens generated by genetic mutations in cancer cells, including those associated with drug resistance [70].

### 9.3. Immune Checkpoint Pathways

Immune checkpoint pathways, such as the programmed cell death protein 1 (PD-1) and cytotoxic T-lymphocyte-associated protein 4 (CTLA-4) pathways, regulate the activity of T cells and their responses to cancer cells. Cancer cells can exploit these pathways to evade immune detection and destruction [71]. Immunotherapies targeting immune checkpoints, known as immune checkpoint inhibitors (ICIs), have revolutionized cancer treatment by rescuing the immune system’s ability to target drug-resistant cancer cells. ICIs block inhibitory signals, allowing T-cells to mount an effective anti-tumor immune response [72].

### 9.4. Tumor-Infiltrating lymphocytes (TILs)

TILs are immune cells that have infiltrated the tumor microenvironment and are actively engaged in recognizing and attacking cancer cells. High levels of TILs, particularly cytotoxic T cells, are associated with better treatment responses and improved outcomes in cancer patients [73]. Strategies to enhance TIL infiltration, such as adoptive cell therapy, aim to reinforce the immune system’s ability to combat drug-resistant cancer cells by increasing the presence of effector immune cells within the tumor [74].

### 9.5. Cancer Vaccines

Cancer vaccines stimulate the immune system to recognize and target cancer-specific antigens, including those associated with drug resistance. Vaccines can lead the immune system to mount a robust and targeted immune response against drug-resistant cancer cells, potentially overcoming resistance mechanisms and improving treatment outcomes [75].

### 9.6. Immunomodulatory Therapies

Besides ICIs, other immunomodulatory therapies, such as cytokine therapy, targeted antibodies, and CAR-T cell therapy, play a critical role in enhancing immune responses against drug-resistant cancer cells. These therapies can activate immune cells, enhance their cytotoxic activity, and modulate the tumor microenvironment to promote anti-tumor immune responses [76].

In summary, the immune system’s role in combating drug-resistant cancer cells is multifaceted and dynamic, involving immune surveillance, cytotoxic immune cell activity, immune checkpoint regulation, TILs, cancer vaccines, and immunomodulatory therapies. Harnessing the immune system’s ability to recognize and target drug-resistant cancer cells holds immense potential in overcoming resistance mechanisms and improving treatment outcomes for cancer patients [77].

### 9.7. Lymphokine-Activated Killer (LAK) Therapy

Lymphokine-activated killer (LAK) therapy exemplifies the critical role of the immune system in combating drug-resistant cancer cells. This immunotherapy approach involves the activation and expansion of a patient’s own lymphocytes, which are key players in the immune response. In LAK therapy, lymphocytes are collected from the patient’s blood and cultured in the laboratory with interleukin-2 (IL-2), a cytokine that stimulates their growth and activity. Once activated and multiplied, these lymphocytes, now known as LAK cells, are reinfused back into the patient to target and destroy cancer cells. The activated LAK cells recognize specific antigens epithelial–mesenchymal transition (EMT) as a target of cancer therapy on tumor cells, bind to them, and release cytotoxic molecules to induce apoptosis [73]. Additionally, the presence of LAK cells and the cytokines they release stimulate other immune components to attack cancer cells. LAK therapy has been explored for treating cancers like melanoma, renal cell carcinoma, and lymphomas. However, its clinical application has been limited due to the high toxicity of IL-2 and variable efficacy. Despite these challenges, LAK therapy underscores the potential of harnessing the immune system to address drug resistance in cancer [73]. While newer immunotherapies such as checkpoint inhibitors and CAR-T cell therapy have largely supplanted LAK therapy due to their more targeted and effective responses, ongoing research continues to explore the integration of LAK therapy with other treatments to enhance cancer treatment outcomes [49,61]. This approach highlights the innovative ways the immune system can be leveraged to overcome drug resistance and improve patient prognosis.

### 9.8. Epithelial–Mesenchymal Transition (EMT) as a Target of Cancer Therapy

Epithelial–Mesenchymal Transition (EMT) is a process where epithelial cells acquire mesenchymal characteristics, increasing their ability to migrate and invade, which is essential for cancer metastasis. Targeting EMT has emerged as a promising cancer therapy approach by inhibiting critical signaling pathways such as TGF-β, Wnt, Notch, and Hedgehog that drive the EMT process. By blocking these pathways, researchers aim to reduce metastasis and enhance the effectiveness of conventional therapies. EMT is also closely linked to drug resistance and immune evasion, making it a significant target for improving cancer treatment. Combining EMT inhibitors with immunotherapies, like immune checkpoint inhibitors, can bolster the immune system’s ability to fight cancer [32,53,61,77]. Ongoing research is focused on developing specific and safe EMT inhibitors, understanding the complex regulation of EMT, and designing effective combination therapies. This approach aims to advance cancer treatment, reduce drug resistance, and ultimately improve patient outcomes through more personalized and effective strategies.

## 10. Unlocking the Power of Immune Therapies: Overcoming Drug Resistance

Immunotherapies, including checkpoint inhibitors and adoptive cell therapies, have emerged as powerful strategies in overcoming drug resistance mechanisms in cancer. These therapies harness the body’s immune system to target and eliminate cancer cells, offering distinct mechanisms that can evade traditional drug resistance [78]. A detailed explanation of how immunotherapies can overcome drug resistance mechanisms is given.

### 10.1. Checkpoint Inhibitors

Checkpoint inhibitors are a type of immunotherapy medication designed to block pathways that cancer cells use to avoid being detected and destroyed by the immune system. One of the most well-known pathways they target is called the PD-1 and PD-L1 pathways. Cancer cells often increase the levels of PD-L1, a protein that binds to PD-1 on T cells. This binding makes the T cells ineffective, leading to immune evasion and exhaustion [79]. Drugs like pembrolizumab and nivolumab target PD-1 or PD-L1, allowing the immune system to better recognize and attack cancer cells, even those that have become resistant to other treatments. Cancer cells can become resistant to chemotherapy or targeted therapy by reducing the molecules or pathways that these treatments target. Checkpoint inhibitors can counter this immune evasion by activating T cells and improving their ability to kill cancer cells, regardless of the specific molecules targeted by other treatments. This expands the range of immune attack and helps overcome resistance caused by changes in molecule levels or pathways [80].

The tumor environment can dampen immune responses against cancer cells, creating a suppressive setting. Checkpoint inhibitors can counter this suppression by revitalizing exhausted T cells and encouraging immune cells to enter tumors [81]. This is especially crucial in cases where drug resistance is linked to immune-suppressing mechanisms like increased regulatory T cell activity or the infiltration of myeloid-derived suppressor cells. Combining checkpoint inhibitors with other immunotherapies, chemotherapy, or targeted treatments is common to boost treatment effectiveness and tackle resistance [82]. For instance, pairing a checkpoint inhibitor with a targeted therapy that boosts tumor antigens or makes cancer cells more vulnerable to immune attacks can synergistically overcome resistance and improve treatment outcomes [83].

### 10.2. Adoptive Cell Therapies (ACT)

Adoptive cell therapies involve manipulating and reintroducing immune cells like T cells or NK cells to target and eliminate cancer cells. One form of adoptive cell therapy, called CAR-T cell therapy, has been incredibly successful in certain blood cancers. In CAR-T cell therapy, T cells are modified genetically to express chimeric antigen receptors (CARs) that can identify and attack specific antigens on cancer cells [84]. This therapy can tackle drug resistance by targeting antigens that traditional treatments do not affect. For instance, in B-cell cancers, CAR-T cells that target CD19, have been proven effective in patients who have not responded to chemotherapy or other treatments. This strategy sidesteps resistance mechanisms linked to changes in drug targets or pathways [85].

Adoptive cell therapies can boost immune cells’ ability to attack drug-resistant cancer cells. Engineered T cells are designed to recognize and destroy cancer cells with precision and strength, leading to lasting responses and overcoming resistance caused by reduced drug sensitivity or drug removal from cells [86]. CAR-T cells and similar therapies can stay active in the body and create memory responses against cancer cells, offering ongoing protection against relapse and recurrence. This is especially helpful in countering resistance related to tumor regrowth after initial treatment [87].

Adoptive cell therapies can also be combined with other immunotherapies like checkpoint inhibitors or cytokine therapies to make them more effective and tackle potential resistance. By targeting different aspects of the immune response, these combined approaches can work together to beat drug resistance and improve treatment results. In essence, immunotherapies, including checkpoint inhibitors and adoptive cell therapies, provide unique ways to overcome drug resistance in cancer [88]. Checkpoint inhibitors stimulate immune responses and reverse immune suppression, while adoptive cell therapies target specific antigens and boost immune cells’ ability to fight drug-resistant cancer cells. Combining these approaches with other treatments can enhance their impact and create comprehensive strategies to fight drug resistance and improve patient outcomes [89].

## 11. Importance of Biomarkers in Predicting Treatment Response and Monitoring Drug Efficacy

Biomarkers are essential tools in modern cancer care, acting as guides for doctors in navigating the complexities of treatment. Their predictive abilities empower oncologists to make informed decisions about the best treatment options for each patient [90]. By examining the genetic makeup of tumors, genetic biomarkers like EGFR or HER2 mutations provide valuable information on which targeted therapies are likely to work best, optimizing treatment outcomes while reducing exposure to less effective treatments [91,92]. Similarly, protein biomarkers like PD-L1 levels help forecast responses to immune checkpoint inhibitors, improving our understanding of how well immunotherapy works in different types of cancer. Functional biomarkers, such as those that assess DNA repair activity, offer predictive hints about a tumor’s sensitivity to specific drugs like PARP inhibitors [93]. This allows for tailored treatment plans that target the molecular weaknesses within cancer cells.

Besides predicting how treatments will work, biomarkers also play a crucial role in keeping track of how effective treatments are over time. Biomarkers from tumors, like circulating tumor DNA (ctDNA) and markers like CA-125, give us real-time updates on how well treatments are working and how the disease is progressing. Changes in these biomarkers show us early signs of whether treatments are effective or if the cancer is resisting treatment [94]. This helps us adjust treatment plans quickly to keep them working well. Pharmacodynamic biomarkers also help us understand how treatments affect the body’s pathways, helping us adjust treatments as needed.

The flexible nature of biomarker monitoring allows us to adjust treatment plans based on how the tumor is changing over time [95]. Identifying resistance biomarkers early on helps us make proactive changes to treatments, trying out different therapies to beat resistance and keep treatments effective [96]. Additionally, markers like minimal residual disease (MRD) help us understand if there is any cancer left after treatment, helping us decide on follow-up treatments or surveillance plans. Overall, biomarkers are incredibly important tools. They not only predict how treatments will work and track their effectiveness but also allow us to adjust treatments as needed, leading to better patient outcomes, higher survival rates, and improved quality of life in cancer care [97]. New biomarkers are changing the way we treat cancer by giving us important information about tumors, how they respond to treatment, and why some drugs stop working. These biomarkers help doctors create personalized treatment plans for each patient, making it easier to beat drug resistance [98]. 

## 12. Initiatives for Therapeutic Approaches in Drug-Resistant Cancers

### 12.1. Understanding Drug Resistance Mechanisms

Researchers are investigating deeply into the mechanisms underlying drug resistance in cancers. This includes studying genetic mutations, epigenetic modifications, tumor microenvironment interactions, and cellular signaling pathways that contribute to treatment resistance. By elucidating these mechanisms, scientists gain crucial insights into potential targets for novel therapeutic interventions [99]. Researchers study mutations in cancer cells that confer resistance to chemotherapy, targeted therapies, or immunotherapy. These mutations can lead to altered drug targets or the activation of survival pathways. Similarly, changes in DNA methylation, histone modifications, or microRNA expression can influence drug response by regulating gene expression [100]. The tumor microenvironment, including immune cells, stromal cells, and extracellular matrix components, also play a crucial role in modulating drug sensitivity or resistance. Dysregulated signaling pathways such as PI3K/Akt/mTOR, MAPK/ERK, and Wnt/β-catenin contribute to drug resistance and are targets for therapeutic interventions [101].

### 12.2. Targeted Therapies and Precision Medicine

Targeted therapies and precision medicine represent a crucial approach. Specific genetic alterations or biomarkers associated with drug resistance are identified, and drugs that selectively target these resistance mechanisms are developed by researchers [102]. This approach is aimed at improving treatment efficacy and reducing side effects by sparing healthy cells. Biomarkers like mutations in EGFR, ALK, HER2, or BRCA genes are used to stratify patients for targeted therapies. Next-generation sequencing and liquid biopsies are utilized for identifying actionable alterations. Similarly, development of small molecule inhibitors that target specific proteins or pathways involved in drug resistance, such as tyrosine kinase inhibitors (TKIs) or PARP inhibitors, is undertaken [103]. Monoclonal antibodies against surface receptors or immune checkpoint proteins are used to enhance targeted killing of cancer cells or restore immune surveillance.

### 12.3. Immunotherapy Advancements

Immunotherapy has emerged as a groundbreaking area in cancer research. Efforts are focused on enhancing immune responses against resistant cancer cells. Strategies include immune checkpoint inhibitors, chimeric antigen receptor (CAR) T-cell therapy, and therapeutic vaccines. Blockade of immune checkpoints like PD-1/PD-L1 or CTLA-4 enhances T-cell activity against tumor cells, overcoming immune evasion mechanisms [104]. Similarly, genetically engineered CAR T-cells recognize and eliminate cancer cells expressing specific antigens, offering a personalized and potent anticancer approach [105]. Therapeutic vaccines targeting tumor-specific antigens or neoantigens stimulate immune responses against resistant cancer cells. These approaches reprogram the immune system to recognize and attack drug-resistant cancer cells [106].

### 12.4. Combination Therapies and Drug Synergy

Combining multiple treatment modalities is another promising strategy. Researchers are exploring synergistic effects between conventional chemotherapy, targeted therapies, immunotherapy, and other interventions. These combinations can overcome drug resistance by attacking cancer cells through multiple mechanisms, reducing the likelihood of resistance development [107]. Combining drugs with complementary mechanisms of action, such as chemotherapy with immunotherapy or targeted therapy, exploits synergistic effects and prevents resistance. Similarly, combining a drug targeting a resistance pathway with another drug that blocks compensatory pathways or enhances immune response can overcome resistance mechanisms [108].

### 12.5. Nanotechnology and Drug Delivery Systems

Nanotechnology plays a significant role in developing innovative drug delivery systems. Nanoparticles can target cancer cells more effectively, bypassing drug resistance mechanisms such as efflux pumps and cellular barriers. Nanoparticles can encapsulate drugs, improving their solubility, stability, and targeted delivery to tumor sites while minimizing off-target effects [109]. Similarly, nano-carriers bypass drug efflux pumps like P-glycoprotein, reducing drug resistance mediated by these transporters. Surface modification of nanoparticles with targeting ligands enables selective binding to cancer cells, enhancing therapeutic efficacy [110]. This improves drug delivery to tumor sites and enhances treatment efficacy while minimizing systemic toxicity.

### 12.6. Artificial Intelligence and Data Analytics

Advances in artificial intelligence (AI) and data analytics are revolutionizing cancer research. Machine learning algorithms analyze vast amounts of data, including genomics, proteomics, and patient outcomes, to identify predictive patterns and optimize treatment strategies. AI-driven precision oncology facilitates personalized treatment plans tailored to individual patients’ molecular profiles [111]. Machine learning algorithms analyze large datasets, including genomic, proteomic, and clinical data, to predict drug response, identify biomarkers, and optimize treatment strategies. AI-driven drug screening identifies existing drugs with potential anticancer effects or synergistic interactions, accelerating drug discovery for resistant cancers. Similarly, AI-based models stratify patients based on molecular profiles, predicting responders and non-responders to specific therapies for personalized treatment approaches [112].

### 12.7. Clinical Trials and Translational Research

Clinical trials play a crucial role in evaluating the efficacy and safety of novel therapeutic strategies. Translational research bridges the gap between laboratory discoveries and clinical applications, facilitating the translation of promising findings into viable treatments for drug-resistant cancers. Collaborations between researchers, clinicians, pharmaceutical companies, and regulatory agencies are essential for advancing these efforts [113]. Adaptive trial designs, incorporating biomarker-driven patient stratification and treatment adaptation based on interim data, optimize clinical trial outcomes, and accelerate drug development. The integration of real-world data from electronic health records, registries, and patient-reported outcomes informs clinical decision-making and validates novel therapeutic strategies outside controlled trial settings. Translating preclinical findings into clinically relevant biomarkers guides patient selection, treatment monitoring, and outcome prediction in clinical practice [114].

In conclusion, ongoing research efforts in developing novel therapeutic strategies for drug-resistant cancers encompass a multifaceted approach, including understanding resistance mechanisms, targeted therapies, immunotherapy advancements, combination treatments, nanotechnology, AI-driven precision medicine, clinical trials, and translational research. These collective endeavors hold immense promise for improving outcomes for patients with challenging-to-treat cancers and driving innovation in oncology [115].

## 13. Challenges and Limitations in Implementing Emerging Therapies in Clinical Practice

Introducing new therapies, especially those targeting drug-resistant cancers, into real-world medical practice comes with challenges and limitations that need addressing to ensure they are widely used and effective. One big challenge is the complexity and variety of cancer itself. Cancer is not just one disease but many, each with different genetic, molecular, and physical traits [116]. This diversity makes it hard to create treatments that work for everyone or to find biomarkers that accurately predict who will benefit from specific treatments. Turning promising lab findings into actual therapies that work in clinics requires thorough testing, tweaking drug formulations, and dealing with regulations, which takes time and resources [117].

Another hurdle is the cost of these new therapies. Advanced treatments like targeted therapies, immunotherapies, and personalized medicine can be expensive due to research, production, and legal costs. This high cost can make it tough for some patients to access these treatments, especially if they do not have good insurance or live in areas with limited healthcare funds. Plus, the tests needed to guide these treatments may not be easy to obtain or be covered by insurance. Keeping up with the fast pace of cancer research and technology is also a challenge [118]. Doctors and other healthcare pros need regular training and support to know about new therapies, testing methods, treatment plans, and how to work together across different specialties. Collaboration among oncologists, pathologists, geneticists, pharmacists, and others is key to making sure these new cancer treatments are used well and have good results [119].

Ethical considerations also come into play when rolling out new cancer treatments. Things like patient consent, privacy, making informed choices, and ensuring fair access to these new treatments need careful thought. It is about finding a balance between the potential benefits of these advanced treatments and making sure patients are safe, have a good quality of life, and achieving good results in the long run. Addressing healthcare disparities is also crucial. This means making sure everyone, especially those in underserved areas or with limited access to healthcare, can obtain these new cancer treatments fairly [120]. Cancer is always changing, and treatments can sometimes stop working or the disease can come back, which creates ongoing challenges. Keeping treatments effective and patients doing well requires keeping a close eye on how patients respond to treatment, spotting resistance early, and changing treatment plans as needed. This means having strong clinical monitoring, checking for biomarkers, and keeping patients involved in their care. Having data over the long term and real-world evidence is key. This helps us see how well these new cancer treatments work, how safe they are, and if they are worth the cost outside of clinical trials [121].

In summary, although new therapies show great potential for changing how we treat cancer and helping patients, bringing them into everyday medical practice comes with many challenges. Solving these challenges needs everyone to work together with researchers, doctors, policymakers, businesses, patient supporters, and healthcare groups. We can tackle these challenges by being open to new ideas, using what we know works, teaching, and learning, making sure treatments are affordable and available to all, thinking about ethics, and using real-world information. These strategies will help us overcome obstacles and make new cancer treatments a normal part of medical care [122].

## 14. Future Perspectives of the Study

The future of overcoming drug resistance in cancer treatment is promising, with ongoing research and innovation poised to significantly enhance therapeutic efficacy. One of the most compelling future perspectives is the potential for drug modification to play a pivotal role in overcoming resistance. Advances in molecular biology, nanotechnology, and computational sciences are expected to converge, leading to the development of more sophisticated and targeted drug modifications. The development of next-generation inhibitors that can specifically target resistance-causing mutations is a key area of focus. These inhibitors are designed to overcome mutations that render first-line treatments ineffective, thereby restoring the sensitivity of cancer cells to therapy. For example, third-generation EGFR inhibitors have shown efficacy against resistant mutations in NSCLC.

**Drug modification strategies:** Nanotechnology will likely play a significant role in drug modification by enabling the creation of nanoparticle-based delivery systems. These systems can enhance the delivery and bioavailability of anticancer drugs, allowing them to bypass mechanisms like drug efflux pumps. Nanoparticles can be engineered to release drugs in a controlled manner, directly at the tumor site, thereby reducing systemic toxicity and improving therapeutic outcomes. The use of prodrugs, which are inactive precursors that convert into active drugs within the cancer cells, offers another innovative approach [109,110]. Prodrugs can be designed to become activated only in the presence of specific cancer cell enzymes, ensuring that the active drug exerts its effect predominantly within the tumor, thereby minimizing resistance and side effects. Modifying existing drugs to include epigenetic components could also be a promising strategy. By incorporating DNA methyl transferase inhibitors or histone deacetylase inhibitors, it is possible to reverse epigenetic changes that contribute to drug resistance, enhancing the efficacy of conventional therapies. Future therapies are expected to leverage combination drug formulations, where multiple agents are combined into a single treatment regimen. This approach can simultaneously target multiple pathways involved in resistance, reducing the likelihood of cancer cells developing resistance to any single agent. Personalized medicine will continue to evolve, utilizing comprehensive genomic and proteomic profiling to tailor drug modifications for individual patients. This approach ensures that treatments are specifically designed to target the unique genetic and molecular landscape of each patient’s cancer, maximizing efficacy, and minimizing resistance [122]. The future will also see the refinement of adaptive therapy regimens that dynamically adjust based on real-time monitoring of tumor response. This adaptive approach can involve modifying drug dosages, schedules, or combinations based on the evolving characteristics of the tumor, thus preemptively addressing the emergence of resistance.

## 15. Conclusions

Exploring new strategies to overcome drug resistance in cancer cells involves a multifaceted approach that includes targeted therapies, advancements in immunotherapy, combination treatments, nanotechnology-based drug delivery systems, and precision medicine guided by biomarker identification [123]. Targeted therapies like small molecule inhibitors and monoclonal antibodies target specific genetic or molecular changes in cancer cells to make treatments more effective. Immunotherapy uses the immune system to identify and destroy drug-resistant cancer cells, including treatments like immune checkpoint inhibitors, CAR T-cell therapy, and therapeutic vaccines [124]. Combining different treatments targets multiple pathways to overcome resistance and improve treatment response. Nanotechnology helps deliver drugs precisely to tumor sites, avoiding drug pumps and improving treatment results. Precision medicine uses biomarkers to create personalized treatment plans based on individual molecular profiles. These different approaches show a variety of ways to overcome drug resistance and improve outcomes for cancer patients [125].

Continued research and collaboration are crucial for advancing cancer treatment outcomes, especially for drug-resistant cancers. Collaboration among researchers, doctors, pharmaceutical companies, regulators, and patient advocates helps drive innovation, speed up drug development, and turn scientific discoveries into practical treatments. Research focuses on understanding drug resistance mechanisms, finding biomarkers, improving treatment combinations, developing targeted therapies, refining drug delivery, using immunotherapy, employing artificial intelligence, running clinical trials, and gathering real-world evidence [126]. This collaborative effort leads to a better understanding of cancer, more precise treatments, improved effectiveness, fewer side effects, longer survival, and a better quality of life for cancer patients. Stressing the importance of ongoing research and collaboration is vital for navigating the complexities of cancer care, addressing treatment challenges, overcoming drug resistance, and ultimately achieving better results in the fight against cancer [127].

## 16. Clinical Impact of Emerging Therapeutic Strategies to Overcome Drug Resistance in Cancer Cells

- Emerging therapeutic strategies, including targeted therapies, combination treatments, and precision medicine, effectively address drug-resistant cancer cells, leading to reduced recurrence rates, minimized side effects, and improved quality of life for patients.
- Innovations in immunotherapies and adaptive strategies expand treatment options, while ongoing research into biomarkers and novel drugs continues to refine and enhance cancer treatment paradigms, ensuring personalized and long-term management of drug-resistant cancers.

## 17. Significance of Emerging Therapeutic Strategies to Overcome Drug Resistance in Cancer Cells

Innovative therapeutic strategies are transforming the battle against drug resistance in cancer cells, improving treatment outcomes and patient care through targeted therapies, combination treatments, and precision medicine. These approaches effectively combat resistance mechanisms, reduce recurrence rates, and minimize side effects, leading to enhanced patient well-being and treatment effectiveness. Advancements in immunotherapies and ongoing research drive the development of new drugs and holistic care models, ensuring a comprehensive and efficient approach to managing drug-resistant cancers.

## Figures and Tables

**Table 1:** A comprehensive overview of the different strategies explored to overcome drug resistance in cancer cells, highlighting the specific approaches and therapeutic interventions for each strategy.

Strategy	Insights	Specific Approaches	Examples of Therapeutic Interventions
**Targeted Therapies**	Focus on specific molecular targets involved in cancer growth and resistance mechanisms.	Development of next-generation inhibitors. Combination therapies targeting multiple pathways. Adaptive dosing strategies to minimize resistance development.	Osimertinib for EGFR T^790^M mutation in lung cancer. Dabrafenib and trametinib in melanoma.
**Immunotherapies**	Harness the immune system to recognize and attack cancer cells and overcome immune escape mechanisms.	Combining ICIs with other therapies. Development of personalized cancer vaccines. Engineering immune cells for enhanced anti-tumor activity.	Nivolumab combined with ipilimumab in melanoma. CAR-T cell therapy targeting CD19 in B-cell malignancies.
**Combination Approaches**	Synergistically target multiple vulnerabilities in cancer cells to prevent resistance development.	Identifying optimal drug combinations Sequencing treatments to prevent resistance. Developing biomarker-driven combination strategies.	Combining PARP inhibitors with platinum-based chemotherapy in BRCA-mutated cancers. Using PD-L1 levels to guide therapy choices.
**Adaptive Therapy**	Dynamically adjust treatments based on tumor response, genetic changes, and disease evolution.	Adaptive dosing and treatment schedules. Treatment interruptions to delay resistance. Sequential and rotational therapies.	Adjusting TKI doses based on real-time monitoring. Planned treatment holidays in hormone receptor-positive breast cancer.
**Genomic and Biomarker-Based Approaches**	Utilize genomic profiling and biomarkers to personalize treatment, predict response, and monitor resistance.	Comprehensive genomic profiling. Liquid biopsies for non-invasive monitoring. Functional biomarkers to guide treatment decisions.	NGS panels for cancer mutation profiling. DNA analysis for emerging resistance mutations. Protein expression levels to select targeted therapies.
**Epigenetic Therapies**	Target epigenetic modifications that contribute to drug resistance.	Use of DNA methyl transferase inhibitors. HDAC inhibitors. Combination of epigenetic drugs with other therapies.	Azacitidine in myelodysplastic syndromes. Vorinostat in cutaneous T-cell lymphoma.
**Metabolic Targeting**	Exploit metabolic vulnerabilities in cancer cells to overcome resistance.	Targeting glycolysis and glucose metabolism. Modulating lipid metabolism. Disrupting amino acid metabolism.	Inhibitors of glycolytic enzymes. Drugs targeting fatty acid synthase (FASN). Inhibitors of glutaminase in glutamine-dependent cancers.
**Cancer Stem Cell (CSC) Targeting**	Address the role of CSCs in drug resistance and tumor recurrence.	Inhibiting CSC-specific signaling pathways. Targeting surface markers on CSCs. Combining CSC-targeting agents with conventional therapies.	Notch inhibitors in CSC-driven tumors. Anti-CD44 antibodies in CSC-enriched populations.
**Nanotechnology and Drug Delivery Systems**	Enhance drug delivery to overcome resistance by improving drug concentration and targeting.	Nanoparticle-based drug delivery systems. Use of targeted drug delivery vehicles. Development of multidrug delivery platforms.	Liposomal formulations of chemotherapeutics. Antibody-drug conjugates (ADCs). Polymeric nanoparticles carrying multiple drugs.

## Data Availability

No new data were created or analyzed in this study. Data sharing is not applicable to this article.

## Abbreviations

ABC, ATP Binding Cassette; ACT, Adoptive CELL Transfer; ADP, **Adenosine Diphosphate**; AI, Artificial Intelligence; **ALK**, Anaplastic Lymphoma Kinase; BCRP, Breast Cancer-Resistant Protein; Bcl-2, B-cell lymphoma 2; Bcl-xL, B-cell lymphoma-extra-large; Bax, Bcl-2-associated-X protein; Bak, Bcl-2 homologous antagonist/killer; BCRA1, Breast Cancer 1; BEP, Base Excision Repair; CDKs, Cyclin Dependent Kinases; CMI, Chronic Myeloid Leukemia; CtDNA, **Circulating Tumor DNA**; **CTLA-4**, Cytotoxic T-Lymphocyte Associated Protein 4; DSBs, Double Stranded Beaks; EGFR, Epidermal Growth Factor Receptor; CAR(T)-cell, Chimeric Antigen Receptor T-Cell; **HER-2**, Human Epidermal Growth Factor Receptor 2; HR, Homologous Recombination; **MAPK**, Mitogen-Activated Protein Kinase; MDR, Multi Drug Resistance; MRI, Magnetic Resonance Imaging; MRD, Minimal Residual Disease; NER, Nucleotide Extension Repair; NSCLC, Non-Small Cell Lung Cancer; PARP, Poly (ADP-Ribose) Polymerase; TKI, Tyrosine Kinase Inhibitors; TIL, Tumor-Infiltrating Lymphocytes.
